# Heterologous Expression of Membrane Proteins: Choosing the Appropriate Host

**DOI:** 10.1371/journal.pone.0029191

**Published:** 2011-12-21

**Authors:** Florent Bernaudat, Annie Frelet-Barrand, Nathalie Pochon, Sébastien Dementin, Patrick Hivin, Sylvain Boutigny, Jean-Baptiste Rioux, Daniel Salvi, Daphné Seigneurin-Berny, Pierre Richaud, Jacques Joyard, David Pignol, Monique Sabaty, Thierry Desnos, Eva Pebay-Peyroula, Elisabeth Darrouzet, Thierry Vernet, Norbert Rolland

**Affiliations:** 1 Institut de Biologie Structurale Jean-Pierre Ebel, CEA, Grenoble, France; 2 Institut de Biologie Structurale Jean-Pierre Ebel, UMR 5075 CNRS, Grenoble, France; 3 Institut de Biologie Structurale Jean-Pierre Ebel, Université Joseph Fourier Grenoble I, Grenoble, France; 4 Laboratoire de Physiologie Cellulaire & Végétale, CEA, DSV, iRTSV, Grenoble, France; 5 Laboratoire de Physiologie Cellulaire & Végétale, CNRS, UMR 5168, Grenoble, France; 6 Laboratoire de Physiologie Cellulaire & Végétale, INRA, UMR 1200, Grenoble, France; 7 Laboratoire de Physiologie Cellulaire & Végétale, Université Joseph Fourier Grenoble I, Grenoble, France; 8 Laboratoire de Biologie du Développement des Plantes, CEA, DSV, iBEB, SBVME, St Paul les Durance, France; 9 Laboratoire de Biologie du Développement des Plantes, UMR 6191 CNRS, St Paul les Durance, France; 10 Laboratoire de Biologie du Développement des Plantes, Université Aix-Marseille, St Paul les Durance, France; 11 Laboratoire de Bioénergétique Cellulaire, CEA, DSV, iBEB, SBVME, St Paul les Durance, France; 12 Laboratoire de Bioénergétique Cellulaire, UMR 6191 CNRS, St Paul les Durance, France; 13 Laboratoire de Bioénergétique Cellulaire, Université Aix-Marseille, St Paul les Durance, France; 14 Laboratoire d'Ingénierie Cellulaire et Biotechnologie, CEA, DSV, iBEB, SBTN, Bagnols-sur-Cèze, France; 15 Laboratoire des Echanges Membranaires et Signalisation, CEA, DSV, iBEB, SBVME, St Paul les Durance, France; 16 Laboratoire des Echanges Membranaires et Signalisation, UMR 6191 CNRS, St Paul les Durance, France; 17 Laboratoire des Echanges Membranaires et Signalisation, Université Aix-Marseille, St Paul les Durance, France; 18 Laboratoire des Transporteurs en Imagerie et Radiothérapie en Oncologie, CEA, DSV, iBEB, SBTN, Bagnols-sur-Cèze, France; University of Cambridge, United Kingdom

## Abstract

**Background:**

Membrane proteins are the targets of 50% of drugs, although they only represent 1% of total cellular proteins. The first major bottleneck on the route to their functional and structural characterisation is their overexpression; and simply choosing the right system can involve many months of trial and error. This work is intended as a guide to where to start when faced with heterologous expression of a membrane protein.

**Methodology/Principal Findings:**

The expression of 20 membrane proteins, both peripheral and integral, in three prokaryotic (*E. coli, L. lactis, R. sphaeroides*) and three eukaryotic (*A. thaliana, N. benthamiana*, Sf9 insect cells) hosts was tested. The proteins tested were of various origins (bacteria, plants and mammals), functions (transporters, receptors, enzymes) and topologies (between 0 and 13 transmembrane segments). The Gateway system was used to clone all 20 genes into appropriate vectors for the hosts to be tested. Culture conditions were optimised for each host, and specific strategies were tested, such as the use of Mistic fusions in *E. coli*. 17 of the 20 proteins were produced at adequate yields for functional and, in some cases, structural studies. We have formulated general recommendations to assist with choosing an appropriate system based on our observations of protein behaviour in the different hosts.

**Conclusions/Significance:**

Most of the methods presented here can be quite easily implemented in other laboratories. The results highlight certain factors that should be considered when selecting an expression host. The decision aide provided should help both newcomers and old-hands to select the best system for their favourite membrane protein.

## Introduction

Membrane proteins (MPs) perform a wide range of essential biological functions and represent the largest class of protein drug targets (for reviews, see [1]–[3]). Approximately 25% of all genes in both prokaryotes and eukaryotes code for MPs [4] and in humans 15% of these are G protein-coupled receptors (GPCRs) [2]. However, the vast majority of MPs still have no assigned function and only a little over 300 unique high-resolution 3D structures have been obtained for transmembrane proteins so far. Most of these structures are for bacterial and archaeal proteins, with only very few from eukaryotic systems [1], [2], [5] (http://blanco.biomol.uci.edu/mpstruc). This does not reflect the efforts deployed for the study of MPs in laboratories worldwide, but is an indication of the technical challenges posed by the hydrophobic nature, generally low natural abundance and intrinsic instability of these proteins. Obtaining sufficient amounts of MPs for functional and structural studies is the first major bottleneck in their study [6]–[12]; and when expressed in heterologous systems, the proteins are frequently *i*) toxic for the host, *ii*) expressed at a very low level in a spatially-delimited membranous environment and *iii*) mis- or unfolded (and thus inactive) [13]. Protein overexpression involves three elements: a gene, a vector and an expression host. The appropriate combination of these elements maximises the amount and quality of protein produced. However, since proteins are very diverse in structure and physico-chemical properties, it is impossible to predict whether a protein of interest will express well, be easy to purify, be active or crystallise in any given experimental setup [14]. Consequently, it is often necessary to test various constructions in diverse expression hosts. Traditional cloning methods with REaL (Restriction Enzyme and Ligase) steps to generate multiple expression plasmids (and constructs) are both labour-intensive and time-consuming. This makes them incompatible with a massively parallel strategy of expression screening. However, over the past few years, several recombinatorial cloning systems have been developed to allow rapid cloning of hundreds of genes and constructs simultaneously [14]–[17]. Among these, the Gateway technology [18], Creator [19] and the fragment exchange (FX) cloning [20] present the advantage of enabling subcloning of an open reading frame (ORF) into multiple expression vectors. Even if often adding extra-sequences to the proteins, Gateway is the most widely used and this technique has already been successfully exploited for high-throughput cloning of MPs [21], and several libraries from various ORFeome projects have been constructed using Gateway vectors [22]–[27]. Gateway technology uses bacteriophage lambda Int/Xis/IHF recombination at *att* sites to transfer ORFs into vectors [28]. This divides the cloning procedure into three steps, as illustrated in Figure S1. In addition, most of the expression vectors available can be made Gateway-compatible by inserting an adapter cassette containing Gateway-specific recombination sites.

Once the expression vectors are obtained, production of the target proteins can be tested in different prokaryotic and eukaryotic expression systems suitable for overexpression of MPs (for reviews, see [12], [15], [29]–[34]). However, each of these systems has pros and cons, and the choice of the appropriate expression system often remains empirical, particularly with regard to the levels of functional protein expression. In the following paragraphs, we will briefly present the host systems tested in this study.

### *Escherichia coli*

*E. coli* is by far the most widely used expression host for the production of recombinant proteins. Its short generation time, low cost and ease of use, as well as its extensive characterisation make it an ideal candidate (for a review see [35]). However, *E. coli* presents some disadvantages for protein overexpression. In particular, many MPs do not fold properly and form aggregates that are then stored in inclusion bodies. Several recent developments have improved the expression of recombinant MPs in *E. coli* [36]. Strains like C41, C43 [13] or Lemo21 [37], which are more tolerant to toxic MPs, or the introduction of tags like GFP [38], MBP, GST, NusA [30] or Mistic [39] can facilitate and improve MP production. Mistic is a 13 kDa protein from *Bacillus subtilis*, which, when produced in *E. coli*, spontaneously associates with the inner membrane, without requiring recognition by the Sec translocon machinery. Due to this spontaneous association with the membrane, Mistic has been successfully used as an *N*-terminal fusion tag to target and facilitate membrane insertion of various cargo MPs in *E. coli* [39]–[45].

### *Lactococcus lactis*

*L. lactis*, like other food-grade lactic acid bacteria, is a non-pathogenic, non-invasive Gram-positive bacterium. These properties have made it a popular candidate for the oral administration of mucosal vaccines (for recent reviews, see [46]–[50]). Thanks to the development of a wide range of genetic engineering tools (for a review see [51]), it is also widely used today for large-scale production of heterologous proteins [29], [30], [46].

Recombinant protein production in *L. lactis* can be performed using the Nisin-Inducible Controlled gene Expression (NICE) system, in which nisin, an antimicrobial peptide, is used to promote the expression of genes positioned in plasmids under the control of the nisin-inducible promoter P_nisA_ (see review [47]). This system has been used to produce various eukaryotic MPs in *L. lactis* [9], [30], [46], [52]–[54]. GFP has also been used to monitor the state of protein folding, in order to select evolved hosts with enhanced functional expression of membrane proteins [55]. One of the major advantages of *L. lactis* over *E. coli* is that inclusion bodies have (so far) not been observed in this host [9]. In addition, it only has a single cell membrane, making the direct use of ligands or inhibitors for activity studies of membrane proteins in whole cells possible. Until recently, expression screening of multiple constructs in *L. lactis* was limited by the absence of efficient cloning procedures, but recent developments based on ligation-independent cloning (LIC) and Gateway technology have made it possible to clone many genes in parallel [54], [56], [57].

### *Rhodobacter sphaeroides*

*R. sphaeroides* is a purple non-sulphur photosynthetic bacterium. The pigment-protein complexes of the photosynthetic apparatus (reaction centres, light-harvesting complexes) are located in invaginations of the cytoplasmic membrane, known as chromatophores. In response to light and/or lowered oxygen tension, the bacteria synthesises large amounts of photosystems [58], and the increasing number of chromatophores causes the membrane surface area to increase vastly. This increase in the intracytoplasmic membrane surface could be very useful for the production of MPs. Indeed, one of the major limitations for MP production in many hosts is the limited membranous space available. In *R. sphaeroides*, foreign MP synthesis can be coordinated with the synthesis of new membranes to accommodate them. This property has already been used to produce heterologous MPs for structural studies [59].

### *Arabidopsis thaliana*

A small flowering plant with a relatively short life cycle of two months, *A. thaliana* is a popular model organism in plant biology and genetics. Its small genome was fully sequenced in 2000 [60]. *A. thaliana* is not regarded as a classical overexpression system since most plant MPs are overexpressed in plants to test their *in vivo* function rather than to obtain sufficient amounts for crystallisation trials. *A. thaliana* can be both stably transformed (by floral dipping [61]) and transiently transformed (by agro-infiltration with *Agrobacterium tumefaciens* [62]). When overexpressing MPs in this organism using stable transformants, the main limitation is the long culture cycle, lasting two months between generations of plant seeds, as compared to only 30 to 50 min for bacteria.

### *Nicotiana benthamiana*

Widely used as an experimental host in plant virology, *N. benthamiana* can be efficiently genetically transformed and regenerated. It is therefore amenable to transient protein expression [63]. This host is rapidly gaining popularity in plant biology, particularly in studies requiring protein localisation, interaction, or plant-based systems for protein expression and purification. Transient *Agrobacterium*-mediated transformation of *N. benthamiana* using leaf disks has provided the plant community with a valuable tool to rapidly evaluate transgenes in higher plants [64] and to produce gram quantities of recombinant proteins [65]. This protocol has a number of significant advantages: readily available explant material, high efficiency, and a relatively quick turnaround time.

### Insect cells and the baculovirus system

The baculovirus system is widely used for eukaryotic protein expression in insect cells [66], [67] as a compromise between bacterial expression and expression in mammalian (stably or transiently transfected) cells. Indeed, although more expensive and time-consuming than expression in *E. coli*, this system is more compatible with eukaryotic proteins because of similar codon usage rules, providing better expression levels and fewer truncated proteins than in bacteria. In addition, this system allows for post-translational modifications. Some of the post-translational modifications produced are not identical to those found in mammals (glycosylations for example), but they are nevertheless closer than those produced by bacteria, or even yeast [68]. Insect cells are easier and cheaper to handle than adherent cells like HEK 293, COS or CHO cells, especially for scale-up. Thus, these cells used with the baculovirus system have a real potential for the heterologous production of MPs. Briefly, the baculovirus system relies on the infection of insect cell lines (usually Sf9, Sf21 or High Five®) by recombinant viruses encoding the gene(s) of interest. Many improvements to recombinant baculovirus generation have been implemented over the last twenty years [69], including the Bac-to-Bac system (Invitrogen), which uses site-specific transposition in *E. coli* rather than homologous recombination in insect cells. Gene expression is generally driven by the polyhedrin or p10 late promoter. A similar system (BacMam, Invitrogen) has recently been developed to allow baculovirus-based expression in mammalian cells.

### Rationale for the current study

Several studies comparing different expression systems for MP production have already been performed. However, these studies focused either on the expression of a given protein [7] or a family of proteins such as GPCRs [12], [70]. Other laboratories have tried to express MPs only in *E. coli* [21], [71], [72] or *L. lactis* [73]. Moreover, except for GPCRs [12], [70], the expression of eukaryotic MPs has only been compared in either prokaryotic [74] or eukaryotic [75] hosts. To our knowledge, our study is the first to compare the overexpression of 20 prokaryotic and eukaryotic MPs in both prokaryotic (*E. coli*, *L. lactis* and *R. sphaeroides*) and eukaryotic (*A. thaliana*, *N. benthamiana* and Sf9 insect cells) expression hosts. This study is also original as we evaluate commonly used hosts such as *E. coli* and, to a lesser extent, insect cells together with more unusual systems, to test their ability to be used as alternative expression hosts. As overexpression of membrane proteins is a challenge in itself, we focused our attention on the production step, and on the yields obtained in the various expression hosts tested. However, in extensions of the present study, we were able to show that some of the proteins produced here could be purified to homogeneity and were active [54], [76], [77]. The present article highlights several successful strategies for the heterologous expression of the MPs studied (from different protein families and with large variations in topology and origin) and discusses possible further improvements to MP expression. But, most importantly, it provides a first-stop analysis of the pitfalls and advantages of the various systems tested depending on the nature of the MP to be expressed. This should be of use to all who are about to venture into this exciting, and sometimes frustrating, field of biology.

## Materials and Methods

### Cloning using the Gateway technology

The cloning steps were performed using Gateway technology (Invitrogen) according to the manufacturer's instructions, but by reducing the volume and quantities of all components (clonase enzyme, buffer, PCR products and vectors) to 1/8^th^ during the recombination steps (BP and LR reactions), to yield a total reaction volume of 2.5 µl that was entirely used for transformation. Briefly, the ORFs coding for the selected proteins (Table 1) were amplified by PCR and flanked with *attB* specific recombination sites. All the genes were also extended with a sequence coding for a *Strep*-tag II at either the *N*- or *C*- terminal end of the constructs. The PCR products were purified and either recombined with a pDONR221 donor vector (Invitrogen) through a BP reaction or cloned into pENTR-D-TOPO vectors through directional topoisomerase-mediated cloning (TOPO, Invitrogen) to yield the “entry” clones. The entry clones were first sequenced to check the integrity of the cloned genes and then used in an LR Gateway reaction together with various destination vectors to yield expression vectors specific to each expression system tested in this study (Table 2).

**Table 1:** List of selected target proteins.

Acc n° UNIPROT	Protein name	Function	Organism	Size (kDa)	Topology^a^	Reference
Q6NCP8	P450	Cytochrome -mono-oxygenase	*R. palustris*	49.7	Peripheral	[87]
O88116	NapC	Cytochrome –electron transfer	*R. sphaeroides*	24.2	1 TM	[110]
Q8DMY2	MreC	Peptidoglycansynthesis	*S. pneumoniae*	29.7	1 TM	[111]
Q8DQH3	FtsX	Cell division	*S. pneumoniae*	34.2	4 TM	[112]
Q8DR69	MraY	Peptidoglycansynthesis	*S. pneumoniae*	36.0	10 TM	[113]
A5X8Y8	LPR1	Multi-copperoxydase	*A. thaliana*	60.5	Peripheral	[114]
Q9SV68	ceQORH	Quinone oxydoreductase –electron transfer	*A. thaliana*	33.1	Peripheral	[100], [101]
Q8GYE0	PHF	Phosphate transport regulation	*A. thaliana*	42.4	1 TM	[115]
Q9M3H5	AtHMA1	Heavy metal transporter	*A. thaliana*	80.1	6 TM	[116]
P31167	AAC	Mitochondria ADP/ATP transporter	*A. thaliana*	33.2	6 TM	[117]
Q66474	AtHMA4	Heavy metal transporter	*A. thaliana*	126.7	8 TM	[118]
Q9SZW5	AtHMA3	Heavy metal transporter	*A. thaliana*	81.4	8 TM	[119]
Q96303	PHT1;4	Phosphate transporter	*A. thaliana*	57.2	12 TM	[120]
Q39002	NTT1	Chloroplast ADP/ATP transporter	*A. thaliana*	57.5	12 TM	[121]
P54290	α2δ subunit	Calcium channel regulation	*R. norvegicus*	122.2	1 TM	[122]
P04633	UCP1	Uncoupling protein	*R. norvegicus*	31.3	6 TM	[123]
Q07817	Bcl-xL	Apoptosis regulation	*H. sapiens*	24.7	1 TM	[124], [125]
P61073	CXCR4	GPCR	*H. sapiens*	37.9	7 TM	[126]
P51681	CCR5	GPCR	*H. sapiens*	38.7	7 TM	[127], [128]
Q92911	NIS	Iodide transporter	*H. sapiens*	67.6	13 TM	[129]

a For some of the proteins, the topology is still unclear and the number of TMs given here corresponds to the predicted topology.

**Table 2:** Protein constructs obtained from the different expression vectors.

Expression host	Expression vector	Expressed protein construct*
*E. coli*	pDEST17	MSYY(H_6_)LE-*att*B1-MP-Strep
*E. coli*	pDEST-Mistic	MSYY(H_6_)LE-Mistic-*att*B1-MP-Strep
*L. lactis*	pNZ8148	MI-*att*B1-MP-Strep
*R. sphaeroides*	pDEST-E	VDI-*att*B1-MP-Strep
*A. thaliana/N. benthamiana*	pAlligator-3	M-MP-Strep
Insect cells	pDEST8	M-MP-Strep

Sequences are presented using one-letter code for amino acids. *att*B1: amino acid sequence encoded by the *att*B1 cloning site corresponding to TSLYKKAGS when the entry clone was prepared though BP cloning and TSLYKKAGSAAAPFT when the entry clone was prepared through TOPO recombination (NapC, P450, LPR1, PHF, PHT1;4, ceQOHR, AtHMA1, Bcl-xL). MP: amino acid sequence of the different membrane proteins. Mistic: amino acid sequence of the fusion tag Mistic. Strep: amino acid sequence of the *Strep*-tag II fusion tag corresponding to WSHPQFEK.

*The *Strep*-tag II was fused to the C-terminus of most proteins, except for proteins AtHMA3, AtHMA4 and Bcl-xL for which the *Strep*-tag II was located at the N-terminus of the MP sequence.

#### *E. coli* expression vectors

To test the expression of the proteins in *E. coli*, the genes were transferred into the destination vectors pDEST17 (Invitrogen) and pDEST-Mistic. pDEST-Mistic was obtained by modifying the vector pDEST17 by introducing the sequence coding for Mistic (Accession n° AAX20121) between the coding sequences of the hexa-histidine tag and the a*tt*B1 site through RF cloning as described by van den Ent and Löwe [78].

#### *L. lactis* expression vector

The vector pNZ8148 containing the NICE system was used for expression in *L. lactis*. This vector wasn't converted into a Gateway destination vector, because it is known to be very unstable in *E. coli* and because of the lack of *Lactococcus* strains able to propagate Gateway vectors. Therefore, the cDNAs were first transferred into the vector pBS-RfA using the Gateway system and subsequently cloned into pNZ8148 through digestion of pBS-RfA vectors by EcoRV and re-ligation (for details, see [54]). For some proteins (MraY, AtHMA3, AtHMA4 and α2δ subunit), with one or several EcoRV restriction sites within the ORF sequence, a partial restriction of the donor plasmids with this restriction enzyme led to a correct excision of the cassette containing the entire gene. Afterwards, *Lactococcus* strain NZ9000 was transformed with the recombinant plasmids as previously described [79] and the presence of the insert in the right orientation was confirmed using restriction analyses, PCR amplification and subsequent sequencing [80].

#### *R. sphaeroides* expression vector

For expression in *R. sphaeroides*, the broad-host-range plasmid pBBR1MCS-2 [81] was modified to convert it into a Gateway destination vector and to change the antibiotic resistance. An omega cartridge encoding resistance to streptomycin and spectinomycin was obtained through BamHI digestion of pHP45Ω plasmid [82] and cloned into pBBR1MCS-2 previously digested with BglII. The *aph* gene encoding resistance to kanamycin was inactivated by the excision of a 400 bp NcoI fragment. To enhance protein expression, the strong promoter and the RBS of the *puc* operon (encoding light harvesting complexes II) were cloned into the resulting plasmid, pBBR1MCSΩ. This was done by amplifying *R. sphaeroides* genomic DNA by PCR, using the primers 5′-AAGGTACCCTGCAGGCCCACGCCCTGAA-3′ and 5′-AAGATATCCACTGTGTCGTCTCCCAACT-3′. The 0.7 kbp PCR product was then digested with KpnI and EcoRV and cloned into pBBR1MCSΩ. Finally, the resulting plasmid was linearised with EcoRV and a Reading Frame Cassette A (RfA) (Invitrogen) was introduced to convert it into a Gateway destination vector.

#### *A. thaliana* and *N. benthamiana* expression vector

The expression vector used for plant transformation was the pAlligator3 vector [83] containing the spectinomycin resistance marker gene and the CaMV 35S promoter (Cauliflower Mosaic Virus). This vector also includes a gene coding for GFP, driven by the At2S3 seed-specific promoter and used as a selectable marker for transformed seeds, as well as the Gateway cloning cassette [83]. *A. tumefaciens* strain (C58) was transformed with the different expression vectors as previously described [84] and the presence of recombinant vectors was verified by plasmid isolation and restriction analysis.

#### Insect cell expression vectors and bacmids

The entry clones were recombined (LR reaction) with the commercial destination vector pDEST8 (Invitrogen) to generate the expression plasmids, which were checked by restriction digest. According to the Invitrogen manual, the only requirement needed to use pDEST8 when designing the “Entry” clone, is the insertion of an ATG start codon for proper initiation of translation. These plasmids were subsequently transformed into DH10Bac™ (Invitrogen) for transposition with the parent bacmid. After the blue/white screening of positive recombinants (LacZα complementation system on the bacmid), the various recombinant bacmids thus obtained were further checked by PCR for the presence of the genes of interest.

### Protein expression in the different systems

#### *E. coli* based expression

Expression vectors were used to transform C43(DE3) (Avidis) and BL21-AI (Invitrogen) competent cells. Expression tests were performed in 24-Deep well plates containing 3 mL of TB medium (100 µg/mL Ampicillin). The cultures were inoculated with overnight pre-cultures at a 1/40^th^ dilution and grown for 2 h at 37°C under agitation (250 rpm). Protein expression was then induced by addition of either 1 mM IPTG for C43 cells or 0.005% (w/v) arabinose for BL21-AI cells and the cultures were incubated for another 16 h at 20°C under agitation (250 rpm). The cells were harvested by centrifugation (3200 *g*, 10 min, 4°C) and the cell pellet resuspended in 250 µL of PBS buffer containing lysozyme (Novagen), benzonase (Novagen) and Complete antiprotease cocktail (Roche). Cells were disrupted using a water bath sonicator and debris were removed by centrifugation (20,000 *g*, 20 min, 4°C). Membranes present in the supernatant were separated by ultracentrifugation (100,000 *g*, 1 h, 4°C). Finally, the membrane pellet was resuspended in 250 µL PBS buffer and 10 µL aliquots were analysed on gradient 4–20% SDS-PAGE gels (Bio-Rad) and by western blots (WB). Total MP content was determined using the BCA protein assay (Pierce).

#### *L. lactis* based expression

Expression tests were performed in 1 L-cultures and crude bacterial membranes were purified as previously described [80]. Briefly, transformed NZ9000 *Lactococcus* cells were grown in 1L of M17 medium (Difco) supplemented with 1% (w/v) glucose and 10 µg/mL chloramphenicol. Cultures were inoculated with 25 mL of overnight pre-cultures and grown at 30°C under gentle shaking (90 rpm). Protein expression was induced when the OD_600_ reached 0.8, with a 0.005 volume of the nisin A-containing supernatant obtained from a culture of the *L. lactis* NZ97000 nisin-producing strain (NIZO). After induction the cells were grown for an additional 4 h at 30°C, under gentle shaking (90 rpm). The cells were then harvested (5000 *g*, 15 min, 4°C) and resuspended in 40 mL of Tris buffer (50 mM Tris-HCl pH 8.0, 100 mM NaCl). Bacteria were lysed using a cell disruptor (One shot, Constant Systems) by 2-fold passages at 35,000 p.s.i. ( = 2.3 kbars) and the lysates clarified by centrifugation (10,000 *g*, 10 min, 4°C). The supernatant containing the membranes was then ultracentrifuged (150,000 *g*, 1 h, 4°C) and the membranes were resuspended in 2 mL of PBS-Glycerol (10% (v/v)). Total MP content was measured using the Bio-Rad protein assay (Bio-Rad) and 20 µg of proteins were analysed on 10% SDS-PAGE and by western blots. Bacteria containing the empty pNZ8148 vector were systematically grown in parallel and used as negative control to validate the nature of the detected signals.

#### *R. sphaeroides* based expression

The expression vectors were mobilised from *E. coli* to *R. sphaeroides* f. sp *denitrificans* IL106 by conjugation. Cells were grown for 24 h at 30°C in Hutner modified medium [85] under aerobic conditions (100 mL medium in 250-mL erlenmeyer flasks, 150 rpm) or phototrophic conditions (180 µmol of photons.m^−2^. s^−1^) with 25 µg/mL kanamycin. Cells were harvested (7000 *g*, 10 min, 4°C) and resuspended in 8 mL of Tris buffer (50 mM Tris-HCl pH 8.0). The bacteria were lysed using a cell disruptor (One shot, Constant Systems) and the lysates clarified by centrifugation (7000 *g*, 10 min, 4°C). The supernatant containing the membranes was then ultracentrifuged (200,000 *g*, 1 h, 4°C) and the membranes were resuspended in 1 mL of Tris buffer. The protein content was measured with the BC assay (Interchim) in 2% SDS, and 25 µg of proteins were analysed by 10% SDS-PAGE and by western blots.

#### *A. thaliana* based expression

Plants were grown in culture chambers at 23°C (8-h light cycle) with a light intensity of 150 µmol·m^−2^·s^−1^ in standard conditions. Wild-type *Arabidopsis* plants ecotype Wassilevskija background were transformed by dipping the floral buds of 4–5-weeks-old plants into an *A. tumefaciens* (C58 strain) solution containing a surfactant (Silwett L-77) according to Clough and Bent [86]. Primary transformant seeds were selected on the basis of GFP fluorescence [83] and germinated in Petri dishes containing solidified medium (Murashige and Skoog, 0.5% (w/v) sucrose, and 0.8% (w/v) agarose) for 2 weeks before transfer to soil. After 3–4 weeks, total MPs were extracted from 1–2 leaves. Finally, membrane proteins were diluted in 200 µL of Tris buffer (50 mM Tris-HCl pH 6.8, 1% Triton X-100) and 25 µg aliquots were analysed on 12% SDS-PAGE and by western blots.

#### *N. benthamiana* based expression

Plants were grown in culture chambers at 20°C (14 h light cycle) with a light intensity of 60–120 µmol·m^−2^·s^−1^ in standard conditions. Three or four week-old wild-type *Nicotiana benthamiana* plants were infiltrated with a solution of *A. tumefaciens* (C58 strain) according to Witte et al. [64]. Total MPs were extracted from 2 leaf discs harvested after 4 days [59]. Finally, membrane proteins were resuspended in 70 µL of buffer (100 mM Tris-HCl pH 8, 5 mM EDTA, 150 mM NaCl, 5 mM DTT, anti protease inhibitors, 1% Triton X-100) and 10 µL aliquots were analysed on 10% SDS-PAGE and by western blots.

#### Sf9 insect cells based expression

The bacmids were amplified in DH10Bac and purified using the S.N.A.P.™ MidiPrep Kit (Invitrogen). Sf9 cells were transfected with cellfectin according to Invitrogen's protocol (Bac to Bac baculovirus expression system) and incubated for 72 h to get the P1 viral stock. This P1 viral stock was then amplified by infecting Sf9 cells and the P2 viral stock thus obtained was subsequently used for expression experiments. The precise titers of these viral stocks have not been determined and after preliminary experiments to determine the best conditions for protein expression, 10% of viral inoculum was used for all the experiments. After infection, cells (approximately 10^6^ per well) were incubated at 27°C for 48 h, and centrifuged for 5 minutes at 1000 *g*. For analyses on whole cell extracts, the cells were then washed in PBS and resuspended in 300 µL of 10 mM Tris-HCl pH 7.4, 150 mM NaCl, 1 mM EDTA, 1% Triton X-100, 0.1% SDS plus protease inhibitors and kept on ice for 20 min. The lysate was centrifuged at 16,000 *g* for 15 min to remove the non-solubilised material. For analyses on membrane fractions, the cell pellet was suspended in 1 mL of cold 20 mM Tris pH 7.5, 250 mM sucrose, plus anti-proteases (Complete, Roche) buffer. After breaking the cells with a Dounce homogeniser (10–15 passages on ice), the lysate was centrifuged at 1000 *g* for 10 min. The supernatant was transferred and centrifuged at 10,000 *g* for 10 min. At last, from this supernatant, membranes were concentrated as a pellet at 100,000 *g* for 1 h. All steps were performed at 4°C or on ice. MPs were diluted in 300 µL of 25 mM Tris, pH 7.5, 100 mM mannitol plus anti-proteases (Complete, Roche). Total MPs were determined using the BCA protein assay (Pierce). For western blot analysis 20 µg of proteins were loaded onto a NOVEX NuPAGE 4–12% Bis-Tris gel (Invitrogen) with MES/SDS running buffer. Non-infected cells were used as a control.

### Western blot analysis

The membrane fraction extracted from cells from each expression system was analysed by western blotting using the *Strep*-tag II sequence as the antigenic epitope, unless specified otherwise. Western blots were performed using the *Strep*-tag HRP Detection Kit (IBA) according to the manufacturer's instructions, unless otherwise stated. The amounts of target proteins present in isolated membrane samples were quantified by densitometry with background correction and comparison to known amounts of a control *Strep*-tagged protein loaded on the same blot. For both plant expression systems we followed the protocol described by Witte et al. [64], with some modifications for *Arabidopsis* by adding a blocking step with the biotin blocking buffer because of the presence of several biotinylated proteins in *Arabidopsis* crude membrane extracts. For *L. lactis*, two different methods were applied depending on the expression level of the protein as previously described [54]. Total membrane protein (TMP) concentrations in isolated membrane samples were also determined using conventional colorimetric methods as stated above.

## Results

### Generation of expression plasmids and cell lines

Our aim was to test the overexpression of 20 MPs (Table 1) in six host organisms, this required engineering 120 expression vectors. Gateway technology was used to optimise and streamline cloning, providing a success-rate over 99% for plasmid generation. The only expression plasmid not produced at all was the *L. lactis* expression vector for the α2δ subunit, which was lost in the cloning step after the Gateway step. This was probably due to the large (>4 kbp) size of the cDNA, or to the presence of several *Eco*RV restriction sites within the gene sequence. In the baculovirus system, all 20 pDEST8 recombinant plasmids were obtained. However, the corresponding bacmids could not be produced for P450 and NIS. In all other organisms, all 20 cell lines were successfully produced.

### Expression results

**Figure 1:**
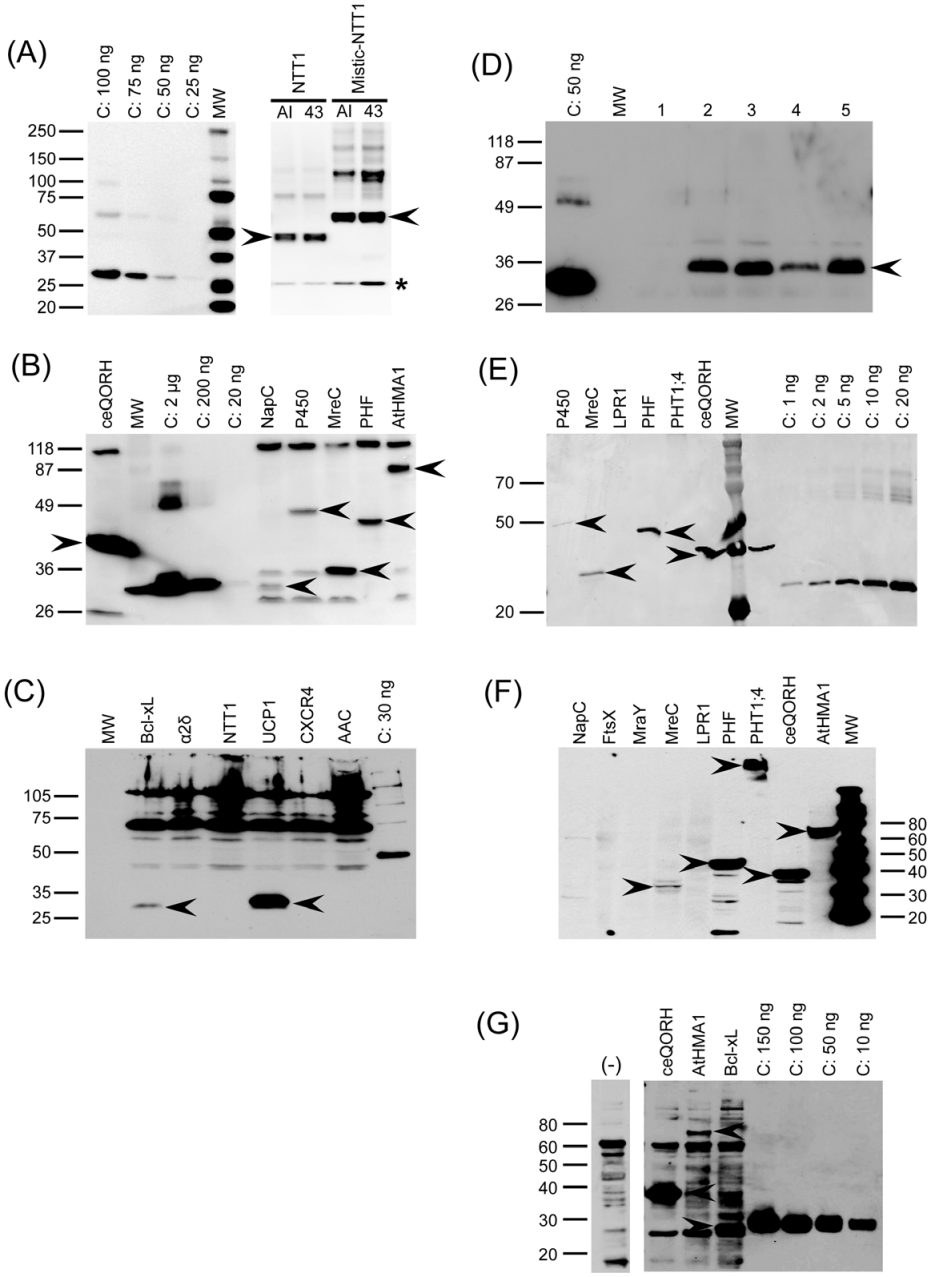
Examples of western blot analysis of cell extracts from the different hosts. (A) **Western blot analysis of membrane extracts of** ***E. coli***. In this case, the native and Mistic-NTT1 fusion. C: *Strep*-tag II control protein loaded at 25, 50, 75 or 100 ng. AI: proteins produced in BL21-AI. 43: proteins produced in C43. MW: molecular weight standard. Arrows point out the different target proteins. *: Partly proteolysed NTT1 protein. The membrane was probed with the *Strep*-Tactin HRP conjugate (IBA). (B) **Western Blot analysis of membrane extracts of** ***L. lactis***. C: *Strep*-tag II control protein loaded at 2000, 200 or 20 ng as written above. MW: molecular weight standard. Arrows point out the different target proteins. The membrane was probed with the *Strep*-Tactin HRP conjugate (IBA). (C) **Western blot analysis of membrane extracts of** ***R. sphaeroides***. C: *Strep*-tag II control protein loaded at 30 ng as written above. MW: molecular weight standard. Arrows point out the different target proteins. The membrane was probed with the *Strep*-Tactin HRP conjugate (IBA). (D) **Western blot analysis of membrane extracts of** ***A. thaliana***. In this case, the expression of the protein AAC was tested in 5 different transformed plants. The membrane fraction was isolated and the extracts corresponding to the different plants tested (lanes 1 to 5) were analysed. C: *Strep*-tag II control protein loaded at 50 ng as written above. MW: molecular weight standard. The arrow points out the protein AAC. The membrane was probed with the *Strep*-Tactin HRP conjugate (IBA). (E) **Western blot analysis of membrane extracts of** ***N. benthamiana*** **leaf discs**. C: *Strep*-tag II control protein loaded at 1, 2, 5, 10 or 20 ng as written above. MW: molecular weight marker. Arrows point out the different target proteins. The membrane was probed with the *Strep*-Tactin HRP conjugate (IBA). (F) **Western blot analysis of whole cell extracts of Sf9 insect cells**. MW: molecular weight standard. Arrows point out the different target proteins. The membrane was probed with the anti-*Strep*-Tag II (IBA) and a goat anti mouse–HRP secondary antibody. (G) **Western blot analysis of membrane extracts of Sf9 insect cells**. This figure is an example of a western-blot for the quantification of target proteins in Sf9 cells membrane vesicles. Here, membrane vesicles of Sf9 cells overproducing either no protein (−), ceQORH, AtHMA1 or Bcl-xL were deposited. C: *Strep*-tag II control protein loaded at 150, 100, 50, 10 ng as written above. Arrows point out the different target proteins. The membrane was probed with the Anti-*Strep*-Tag II (IBA) and a goat anti mouse–HRP secondary antibody.

The proteins in this study belong to diverse protein families, are of both prokaryotic and eukaryotic origin, and their topology ranges from peripheral MPs to integral MPs (IMPs) containing between one and thirteen predicted transmembrane (TM) regions (Table 1). To evaluate the efficiency of the different expression systems, after protein expression, the membranes were isolated as described in Material and Methods. The amount of target protein in the membranes was determined by western-blot, using the *Strep*-tag II sequence (if not otherwise stated) to reveal the presence of target protein on the membrane (See Figure 1 (A) to (G) and Table 3). Expression levels are generally given in mg/L of culture for bacteria and Sf9 cells. However, because we also used plant systems, we also considered the production levels as a percentage, target MP within the total pool of membrane proteins (TMP) (Table 3). This made it possible to compare all the different expression systems used here.

**Table 3:** Protein yields obtained in the different expression hosts.

Protein name	*E. coli* (His)	*E. coli* (Mistic)	*L. lactis*	*R. sphaeroides*	Insect cells	*A. thaliana*	*N. benthamiana*
P450	♦♦♦♦ (5–10%)	–	♦♦ (1–3%)	♦♦♦♦ (1–3%)	n.c.	n.a. (<0.1%)	n.a. (<0.1%)
NapC	♦♦♦ (3–5%)	♦♦♦♦ (15–20%)	♦♦ (0.5–1%)	–	♦ (<0.1%)	n.a. (0.1–0.5%)	n.a. (<0.1%)
MreC	♦♦♦♦ (10–15%)	♦♦♦♦♦ (15–20%)	♦♦♦ (0.5–1%)	–	♦ (<0.1%)	n.a. (<0.1%)	n.a. (<0.1%)
FtsX	♦♦♦ (3–5%)	♦♦♦♦ (5–10%)	–	–	–	–	–
MraY	–	–	–	–	–	–	–
LPR1	♦♦♦♦ (1–3%)	–	♦ (0.1–0.5%)	–	–	–	–
ceQORH	♦♦♦♦♦♦ (15–20%)	♦♦♦♦ (10–15%)	♦♦♦♦♦♦ (30%)	♦ (<0.1%)	♦♦ (0.5–1%)	n.a. (0.1–0.5%)	n.a. (0.5–1%)
PHF	♦♦♦♦♦ (5–10%)	♦♦♦♦♦ (10–15%)	♦♦♦♦ (1–3%)	–	♦♦ (0.1–0.5%)	–	n.a. (0.1–0.5%)
AtHMA1	–	♦♦♦ (3–5%)	♦♦ (1–3%)	–	♦ (<0.1%)	n.a. (<0.1%)	–
AAC	♦♦♦ (1–3%)	♦♦♦♦ (3–5%)	–	–	♦♦ (0.1–0.5%)	n.a. (0.1–0.5%)	–
AtHMA4	–	–	♦ (0.5–1%)	–	–	–	–
AtHMA3	–	–	♦♦ (0.5–1%)	–	–	n.a. (<0.1%)	–
PHT1;4	♦♦♦ (3–5%)	♦♦♦♦ (5–10%)	–	–	–	–	–
NTT1	♦♦♦♦ (3–5%)	♦♦♦♦♦ (5–10%)	♦♦ (0.1–0.5%)	–	–	n.a. (<0.1%)	–
α2δ subunit	–	–	n.c.	–	–	–	–
UCP1	♦♦♦ (3–5%)	♦♦♦♦ (5–10%)	–	♦♦ (0.1–0.5%)	♦♦ (0.1–0.5%)	n.a. (<0.1%)	n.a. (<0.1%)
Bcl-xL	♦♦♦ (1–3%)	♦♦♦♦ (3–5%)	♦♦ (0.5–1%)	♦ (<0.1%)	♦♦ (0.1–0.5%)	n.a. (<0.1%)	n.a. (<0.1%)
CXCR4	♦♦♦ (1–3%)	♦♦♦♦ (3–5%)	♦ (<0.1%)	–	–	–	–
CCR5	♦♦♦ (1–3%)	♦♦♦♦ (3–5%)	♦ (<0.1%)	–	–	–	–
NIS	–	–	–	–	n.c.	–	–

For each protein, the yield obtained is expressed in mg of target protein/liter of cell culture (black squares) and as percentage of total membrane protein (% TMP) (figures in brackets). –: protein not detected by western blot, ♦ = protein produced at a yield below 0.1 mg/L culture, ♦♦ = 0.1–0.5 mg/L, ♦♦♦ = 0.5–1 mg/L, ♦♦♦♦ = 1–4 mg/L, ♦♦♦♦♦ = 4–7 mg/L and ♦♦♦♦♦♦ >7 mg/L. n.a. = not applicable, as the two plant systems are not cultured in liquid media. n.c. = not cloned.

#### Expression in *E coli*

**Figure 2:**
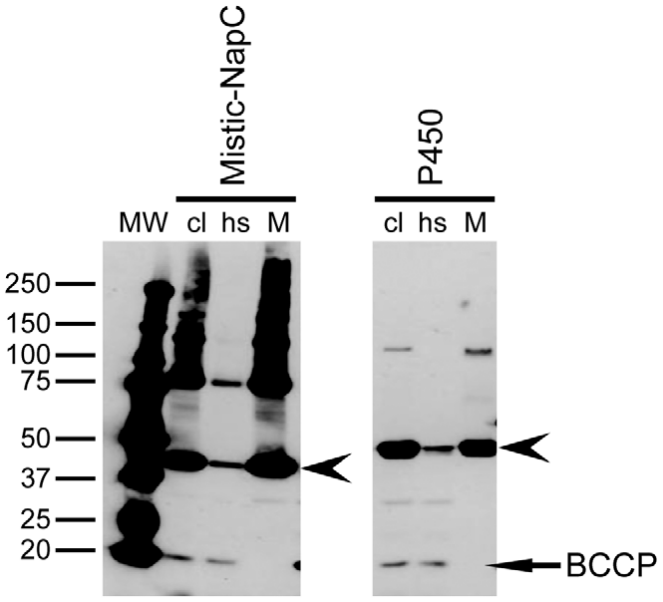
Isolation of the membrane fraction from *E. coli* cells. In this case extracts of *E. coli* cells overexpressing either the Mistic fusion of the protein NapC or the protein P450. cl stands for “cleared lysate” corresponding to the supernatant recovered after centrifugation, at 20,000 *g*, of the cell lysate. hs and M stand for “high-speed supernatant” and “membrane fraction”, respectively, corresponding to the supernatant and the resuspended pellet recovered after ultra-centrifugation, at 100,000 *g*, of the “cleared lysate”. Arrows point out the different target proteins and the endogenous *E. coli* biotinylated protein BCCP. The membrane was probed with the *Strep*-Tactin HRP conjugate (IBA).

Prior to the screen of the 20 proteins, several expression conditions (concentration of inducing agent and temperature) were tested for the production of a few proteins in BL21-AI. A concentration of 0.005% arabinose and an overnight induction at 20°C gave the best results. For C43 strain, a concentration of 1 mM IPTG was retained. These conditions were then applied in the expression screening that was performed in triplicate for all proteins in both strains. No significant differences were observed between the strains in terms of expression levels, therefore the results were averaged in Table 3. Two plasmids were used to transform *E.* coli: pDEST17 yielded a construct in which the amino acids encoded by the *att*B1 recombination site formed a linker between an *N-*terminal His-tag and the proteins (Table 2); whereas with pDEST-Mistic, Mistic was located between the *N*-terminal His-tag and the *att*B1sequence, followed by the target protein (Table 2). A representative western blot of proteins produced in *E. coli* is shown in Figure 1A. Detection of western blots using *Strep*-Tactin HRP conjugates had a useful side-effect in *E. coli,* where a soluble endogenous biotinylated protein, biotin carboxyl carrier protein (BCCP; 22.5 kDa), was detected. This protein should be absent from the membrane fraction and was therefore used to control the purity of this fraction (Figure 2).

Detectable amounts of full-length protein were obtained for 15 out of the 20 MPs (the three peripheral proteins and 12 IMPs) in *E. coli* with or without fusion to Mistic. For the other five proteins, either no signal was detected, or the MW was too far removed from the expected value (e.g. for NIS, a signal was observed at one third the expected MW). Bands of this type could be the result of proteolytic degradation, internal initiation or premature termination. Since the proteins produced in *E. coli* had both a His-tag and the *Strep*-tag II sequence, western blots could also be probed using anti-His antibodies. This was done for a few proteins that were not detected with the *Strep*-tag, to check whether the lack of signal was due to the absence of the protein, or to a tag detection problem. For FtsX, a signal was indeed obtained with anti-His, indicating that, for this protein there was some problem with the *Strep*-tag II. This type of problem may also have occurred for some proteins in the other expression systems (see below).

Mistic fusion significantly increased the yields of the 12 IMPs produced in *E. coli*. In contrast, it had a negative effect when fused to peripheral proteins, drastically reducing the amount of target protein associated with the isolated membranes in all three cases. This should therefore be taken into account when selecting a vector for protein expression.

Functional studies, detailed elsewhere [54], [76], [77], showed several of these proteins to be active and readily purified.

#### Expression in *L. lactis*

**Figure 3:**
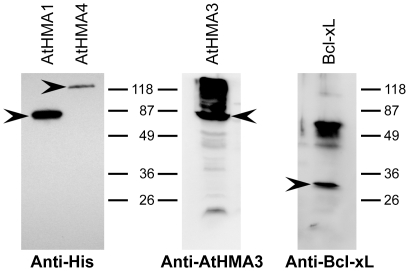
Particular cases of proteins detected in western blots using specific antibodies. For the detection of AtHMA4 by anti-His antibodies, AtHMA1 was also added on the blot as a positive control. Arrows point out the different target proteins.

Before screening for expression of all proteins in *L. lactis*, culture conditions were optimised (temperature, induction time and concentration of nisin) for two representative proteins, one peripheral (ceQORH) and one intrinsic MP (AtHMA1). The nisin used for induction was produced in-house as described in Material and Methods, and the same batch was used for all the tests performed in this study. Optimal production of both proteins was achieved by adding 0.005 volume nisin A-containing NZ9700 medium supernatant to a culture at OD600≈0.8. Production levels were two- to three-fold higher when the cells were grown at 30°C for 4 h after induction, rather than overnight at 20°C (data not shown). These culture and induction conditions were then applied to test the expression of all 20 proteins. Thirteen of the 20 proteins tested were successfully produced. The quantities of MPs obtained were about 1/10^th^ those provided by *E. coli* (Table 3; for a representative western blot, see Figure 1B). However, the plant protein ceQORH was produced at 9 mg/L, which corresponded to 30% of the TMPs in these cells. This is comparable to the levels obtained with prokaryotic MPs. As in *E. coli*, some of the proteins that could not be identified through the *Strep*-tag II, could be detected using other antigenic epitopes. For example, AtHMA4, which contains an internal poly-histidine sequence, could be detected using anti-His antibodies, while AtHMA3 and Bcl-xL were detected using protein-specific antibodies (Figure 3).

Further functional studies were performed on some of the proteins expressed in *L. lactis*, these are detailed elsewhere [54]. The specific activity of the protein ceQORH was significantly improved in this host compared to *E. coli*.

#### Expression in *R. sphaeroides*

In *R. sphaeroides*, intracytoplasmic membrane is synthesised in response to specific growth conditions. We tested the expression of target proteins under both phototrophic anaerobic conditions and semi-aerobic conditions. The different conditions did not have a significant impact on results, and only four proteins could be produced in this host (Table 3; for a representative western blot, see Figure 1C). Cytochrome P450 was found to be correctly folded and active, since it could fix CO [87]. However, we were quite surprised by the limited (20%) success rate of membrane protein expression using this system. Indeed, in other experiments, large amount of soluble proteins were produced using either a pRK415 [88] or a pBBR1MCS-2 derivative with the *puc* promoter. This vector also allows expression of MP, since we were able to express cytochrome *bc1* and to complement a cytochrome *bc1* null mutant (data not shown). Even more surprising was the lack of production of homologous NapC, the tetraheme electron donor of the periplasmic nitrate reductase, NapAB. To test whether this was due to the use of the Gateway cloning approach, we cloned the *napC* gene in the pBBR1MCS2 vector with the *puc* promoter using traditional cloning methods (Restriction enzyme and Ligase). This plasmid was able to restore the nitrate reductase activity in a *R. sphaeroides napC* null mutant, thus demonstrating effective expression of active NapC. This indicates that, with the Gateway expression system, the additional amino acids encoded by the *att*B1 sequence (Table 2) may be a source of problems in this host. Alternatively, as we did not perform functional studies with the Gateway clones, the absence of protein detected by western blot could simply be the result of problems with *Strep*-tag II detection, as mentioned above for other proteins, rather than low expression or absence of product in this system. Whatever the case, this host is not an ideal candidate for Gateway-based protein expression of MPs in the conditions used in this study.

#### Expression in *A. thaliana*

**Figure 4:**
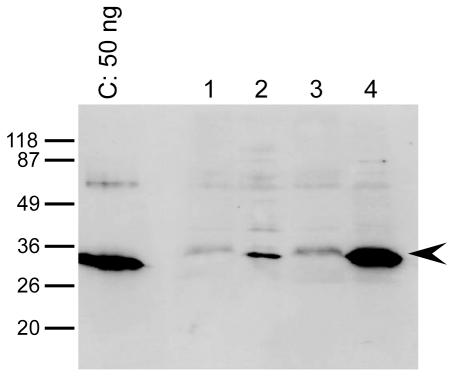
Homologous production of AAC in *A. thaliana* and presence of the recombinant protein in its original organelle, revealed by western blot. Mitochondria were isolated and enriched, from the leaves of 8 weeks old heterozygous *Arabidopsis* plants overexpressing the protein AAC, according to two isolation methods described by Keech et al. (lane 2) or by Brugière et al. (lane 4). Lanes 1 & 3 total membrane extracts before the mitochondria isolation treatments. C: *Strep*-tag II control protein loaded at 50 ng. The arrow points out the protein AAC. The membrane was probed with the *Strep*-Tactin HRP conjugate (IBA).

In *A. thaliana*, 50% of the proteins tested were produced (Table 3, for a representative western blot, see Figure 1D). Surprisingly, not all the proteins originating from Arabidopsis were successfully expressed. This may be due to a detection problem, as discussed above. Alternatively, it may stem from silencing of the transgene by the host cells. For example, all the plants carrying the *LPR1* transgene displayed a typical *lpr1* mutant phenotype, indicating that the endogenous *LPR1* gene was also silenced (data not shown). The ubiquitous presence of biotinylated proteins in *A. thaliana* made it necessary to adapt conditions for the western blot analysis, such as by the use of specific blocking buffer and/or avidin prior to conjugate incubation, as described by Witte et al. [64]. As several Arabidopsis proteins were tested in this study, in some cases we checked whether the recombinant protein was correctly targeted to its native location (membrane). For instance, AAC is an ADP/ATP transporter located in the inner mitochondrial membrane. Mitochondrial membranes were purified from the leaves of transgenic Arabidopsis plants overexpressing AAC using two different isolation procedures [89], [90]. The presence of the recombinant protein was assessed by western blot using the *Strep*-Tactin HRP conjugate (Figure 4). While we found the procedure described by Brugière et al. [89] to be more efficient, in both cases the protein was enriched in the mitochondrial fraction compared to crude cell membranes, demonstrating efficient targeting of the recombinant protein to the organelle and indicating that the *Strep*-tag II did not interfere with its insertion into the membrane.

#### Expression in *N. benthamiana*

Seven proteins were successfully expressed in *N. benthamiana* (Table 3), as shown for five of them in Figure 1E. These proteins are mainly peripheral proteins or proteins with only one predicted TM domain. A single protein with more than one predicted TM (UCP1 = 6 TM) was successfully produced. ceQORH was particularly well expressed in this system, to levels detectable on Coomassie gels (data not shown). All but one (PHF) of the proteins detected in *N. benthamiana* were also produced in *A. thaliana*, indicating that both hosts can be used almost equally successfully.

**Figure 5:**
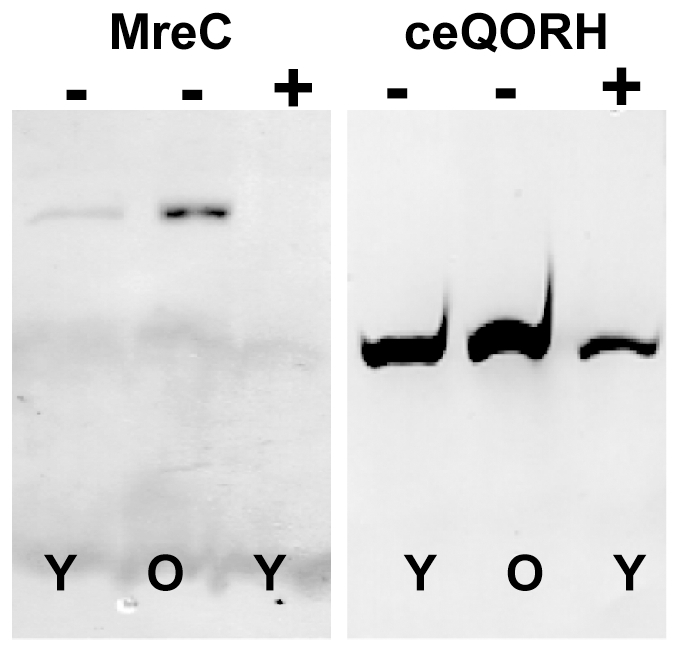
Effect of plant age and light intensity on the expression of MreC and ceQORH in *N. benthamiana.* The *N. benthamiana* plants were grown under low light (60–120 µE) or high light (240 µE) before the infiltration with *Agrobacterium*. The young plants had 4 to 6 leaves whereas the old plants started to blossom. The membranes were then extracted and 6.8 µg of total proteins were loaded on a gel and western-blotted. Y: young plant; O: old plant; (−): light intensity of 60–120 µE; (+): light intensity of 240 µE. The membrane was probed with the *Strep*-Tactin HRP conjugate (IBA).

During optimisation of our experimental set-up for the overexpression of MPs in *N. benthamiana*, we observed that two conditions significantly influenced the levels and/or stability of several recombinant proteins: the growth stage of the plant, and the light intensity in the growth chamber. The expression levels of the recombinant MreC and ceQORH, in variations on the above-cited growth conditions, are shown in Figure 5 as examples of the effects of these parameters. Both MreC and ceQORH accumulate more in older plants than in young ones. Increased accumulation was also observed when young plants were grown under low light. Although we did not perform any further experiments to elucidate this phenomenon, it is possible that, since high light can induce oxidative stress in *N. benthamiana* [91], this may depress protein synthesis and/or accumulation.

#### Expression in Sf9 cells

As mentioned above, recombinant bacmids were obtained for 18 genes. A first set of expression tests was analysed by western blot on total extracts from cells infected with these bacmids (see an example in Figure 1F). Twelve proteins could be detected in whole-cell extracts, and their expression was also analysed on membrane vesicles (Figure 1G). The other 6 proteins (FtsX, MraY, AtHMA4, AtHMA3, α2δ subunit, and CXCR4) were undetectable. Among the proteins expressed, AAC showed variable results between expression experiments, and even over time with the same sample. This suggests significant protein instability. NapC and LPR1, although expressed were mainly present in the unsolubilised material (see Materials and Methods); while PHT1;4, NTT1 and CCR5 all migrated at very high molecular weights, suggesting that they were aggregated (see Figure 1F for PHT1;4). Some of the proteins produced in this host were difficult to quantify for reasons including: very high background staining on western blots with membrane vesicles (NapC); poor transfer of aggregated protein forms (PHT1;4, NTT1 and CCR5), and; extremely low presence in the purified membrane fraction (LPR1). Taking all this into consideration, eight out of 18 proteins were correctly expressed at levels ranging from around 10–20 µg/L of culture, for AtHMA1, NapC and MreC, to 330 µg/L, for ceQORH; Bcl-xL, PHF, UCP1 and AAC were produced at intermediate levels (Table 3). Although these levels are quite low compared to those produced in bacterial cultures, as mentioned above insect cells have certain advantages when expressing eukaryotic proteins, such as the ability to insert post-translational modifications and disulfide bridges. Thus, in functional tests, these cells might be a better choice.

## Discussion

### Gateway vectors for cloning and expression

The cloning strategy chosen for this project, based on Gateway technology, enabled us to obtain expression vectors for the different systems in a convenient and very efficient manner (over 99% success). Adaptation of the manufacturer's protocol by an 8-fold reduction of the volumes and quantities of the components used in the BP and LR reactions also enabled us to significantly reduce the cost of the cloning with a maintained efficiency. However, Gateway does present some disadvantages; in particular, the specific *att*B recombination sites used for cloning introduce additional amino sequences at the *N*-terminus of the recombinant proteins. Because we decided to use the same “entry” clones for all the expression systems, for expression in bacterial systems, the Ribosome Binding Site (RBS) necessary for the translation as well as the initiation codon had to be present in the destination vector upstream of the *att*R1 sequence. After the LR Gateway reaction, the expression vector codes for a protein that contains 12 to 18 additional residues at its *N*-terminus (Table 2). Although short, this additional sequence has a net charge which could interfere with membrane insertion of the target proteins. A previous study demonstrated that shortened *att* recombination sites increased the success rate for MP expression in *E. coli* [72]. However, the influence of these extensions appears to be variable, depending on the topology of the tested proteins [92]. Indeed, in this study expression in *E. coli* was highly successful using this strategy (15 proteins out of 20 detected in isolated membranes). In a previous study, we showed that the presence or absence of these sites did not affect the level of MP production in *L. lactis* [54]. However, the presence of these additional residues could affect expression in other bacterial hosts and perhaps explains the lower rate of success in *R. sphaeroides*. In mammalian cells, adding this extra sequence at the *N*-terminus of NIS protein has quite a negative effect, worse than the absence of a Kozak consensus sequence (data not shown). The addition of a Flag-tag epitope to the *N*-terminus of NIS also hampers its expression (yields, maturation) [93]. Because of these potential problems with protein expression, the constructs for expression in insect cells were designed not to contain the *att* sequence within the expressed protein. Among recombinatorial cloning methods, only MAGIC [94] and In-Fusion [95] enable seamless cloning, but these two methods require independent PCR products for every new construct and are thus not readily compatible with high-throughput approaches. A recent work by Geertsma and Dutzler [20] presents an elegant new system termed fragment exchange (FX) cloning, which enables subcloning into multiple expression vectors and introduces only a single amino acid to either side of the protein. FX cloning will most probably become very popular in a near future, but for the time being, no compatible vectors are yet commercially available and plasmids need to be constructed and adapted to the technique. To conclude on the cloning strategy, given the efficiency of the cloning step and the number of ORFeome projects (and thus the huge number of readily available entry clones) for which Gateway technology has been successfully exploited (for some recent examples see [24]–[27]), we recommend its use when cloning a large number of target genes in various vectors.

### *Strep*-tag II to reveal protein expression

In this study, the 20 proteins were labelled with a *Strep*-tag II sequence (Trp-Ser-His-Pro-Gln-Phe-Glu-Lys). This tag binds strongly to an engineered streptavidin derivative called *Strep-*Tactin. It enables fast and simple one-step purification, and is compatible with a wide range of detergents commonly used for the solubilisation of membrane proteins [96]. Comparison of a range of affinity tags to purify recombinant proteins from various cell types also revealed that the *Strep*-tag offered the best compromise in terms of purity and costs [97]. In our case, the tag was mainly used for target detection in western blotting, to determine the expression level of the proteins. The main reason why we chose *Strep*-tag II over the more commonly used His_6_-tag is that a large number of plant proteins contain natural polyhistidine sequences that could lead to false positive responses with anti-His antibodies. This would also hinder the detection of poorly expressed proteins. In addition, commercial anti-*Strep* HRP-conjugates or antibodies were available, together with protein standards for blot calibration in all the laboratories involved in this project. Despite all these arguments in favour of *Strep*-tag II, analysis of overexpressed proteins in *A. thaliana* and Sf9 cells was difficult because a large number of endogenous biotinylated proteins were present and revealed by the *Strep*-Tactin conjugates used for detection in western blots. In these systems, additional blocking steps were required, to saturate the biotinylated proteins with avidin. In other systems, however, these endogenous biotinylated proteins were an advantage. For example, in *E. coli*, the biotinylated BCCP protein was used to control for membrane isolation (Figure 2) while, in *L. lactis,* several endogenous membrane proteins are biotinylated and could be used as protein loading controls for western blots (Figure 1B).

The topology of some proteins in this study is still unknown, and could be modified when they are produced in heterologous systems. We could not therefore predict which protein extremity would be cytoplasmic. This was another reason to avoid using polyhistidine tags, which can be positively charged at physiological pHs. A positive charge is not theoretically favourable to insertion in, or passage through, membranes. In contrast, the *Strep*-tag II is neutral [92]. Despite this, all the constructs expressed in *E. coli* had an *N*-terminal His_6_-tag and this had no apparent deleterious effect given the high success rate and protein yields in this host. However, it is possible that these yields could have been further improved if the tag had not been included.

We do not know whether the failed detections in western blots were due to an absence of protein expression, or to loss or inaccessibility of the *Strep*-tag II. In several cases (FtsX, AtHMA3, AtHMA4, Bcl-xL) the protein was detected using other antigenic epitopes (anti-His or protein-specific antibodies) (Figure 3). Therefore, it should be kept in mind that failed detection does not necessarily indicate failed expression. To avoid this type of problem, it is advisable to use different methods of detection, or to modify the protein construct, for example, by moving the tag to the other extremity, or adding extra linkers between the tag and the protein. In another study, modified constructs of AtHMA3 and AtHMA4 which placed the *Strep*-tag II at the *C*-terminus were engineered for expression in *L. lactis*. These proteins were detected normally using *Strep*-Tactin [54]. However, in this parallel screening procedure, it was necessary to limit the number of constructs to be tested (already 120) by making choices, these may cause our results to appear poorer than they are in reality.

### Influence of protein properties on expression

**Figure 6:**
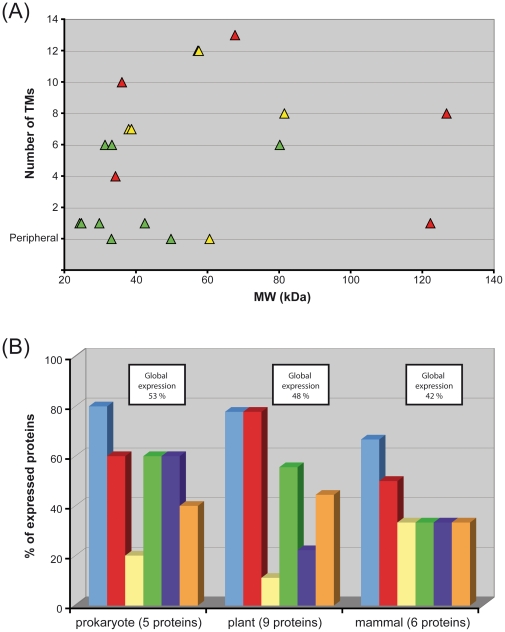
Influence of protein properties on expression. (A) **Influence of the protein size and the number of TMs on the expression success rate.** The triangles represent each proteins and their colour the success with which they were expressed in the different expression systems. Red = protein expressed in none or only one of the expression systems. Yellow = protein expressed in two or three of the expression systems. Green = protein expressed in four to six of the expression systems. (B) **Influence of the origin of proteins on the expression in the different systems.** The bars represent the percentage of positively expressed proteins in each expression host for a given category. Light blue: *E. coli*; Red: *L. lactis*; Yellow: *R. Sphaeroides*; Green: *A. thaliana*; Dark blue: *N. benthamiana*, Orange: insect cells. Global expression represents the percentage of positively expressed proteins in all expression hosts for a given category.

Six expression systems, three prokaryotic (*E. coli, L. lactis* and *R. sphaeroides*) and three eukaryotic (*A. thaliana, N. benthamiana* and Sf9 cells), were evaluated for their ability to overexpress a set of 20 MPs in this study. Besides their scientific importance, the different proteins were selected to cover a broad range of protein families, source organisms, topologies and functions. Of the 20 MPs, 17 (85%) were produced in at least one of the expression hosts; at levels compatible with further functional and even structural studies in some cases (70%>1 mg/L). However, as shown in Table 3, the expression results were extremely variable. Proteins with a large number of predicted TMs or a large MW were generally less successful (Figure 6A). However, the trend in individual systems sometimes differed from the global picture (Figures S2 and S3). All the peripheral, and most IMPs containing between one and six predicted transmembrane helices, were successfully overexpressed. However, for IMPs with higher numbers of TMs, the outcome was more random, and these were often not expressed at all, which is consistent with previous studies [98], [99]. The size of the proteins is expected to affect their expression, as smaller proteins necessarily contain fewer TMs, and in our selection all seven proteins of less than 35 kDa contain a maximum of six predicted TMs. These two parameters could explain why some proteins were (or were not) expressed in all the systems tested. For instance, ceQORH was produced in high quantities in all systems. It is a peripheral, rather hydrophilic protein, and, as previously demonstrated [54], [100], [101], only interacts with the membrane through electrostatic interactions. Bcl-xL, contains only one predicted TM, and was also well expressed. On the other hand, among the proteins not expressed, “α2δ subunit” has only one predicted TM but a molecular weight over 120 kDa, while MraY and NIS are predicted to have 10 and 13 TMs respectively.

The organism of origin of the protein (including eukaryote versus prokaryote) did not appear to have a significant influence on the efficiency of expression (Figure 6B). Heterologous expression was often successful, and homologous expression sometimes failed, as in the cases of *R. sphaeroides* and *A. thaliana*.

When working with large and/or highly hydrophobic MPs, none of the hosts tested really stood out, with maybe a small exception for *L. lactis*, but the statistics are too small to really conclude. With these proteins one should thus expect that successful expression will require more effort and should focus on optimising the expression conditions (level and time of induction, temperature, additive in the growth medium like glycerol or sucrose…). For example, in the work by Wagner et al. [37] describing the *E. coli* strain Lemo21(DE3), the authors clearly demonstrated that variations of growth conditions could significantly impact on the levels of expression. In a parallel screening, choices need to be made and the number of growth conditions tested is limited. In this study, prior to the screen, several conditions were tested in the different hosts, with one or more proteins to define standard expression conditions that were finally used for all proteins. These conditions were therefore most probably not optimal in all cases. Then, after identification of the most suitable expression system for one candidate protein, it may also be required to further optimise specific expression conditions.

### Mistic, a boost in *E. coli* expression

Overall, the best expression results in terms of success rate and protein amounts were obtained with *E. coli* (protein yields >1 mg protein/L culture for 14 proteins). Many possible fusion tags or proteins such as GST, MBP, NusA or Mistic [30], [39] have been described in the literature, however no system is perfect and can solve all the problems. Mistic seems to act like a signal sequence that targets the proteins to the inner membrane of *E. coli*. Its properties and the good results obtained by others with Mistic fusions [39], [40], [43], [45] determined our choice to use it in this study. In our hands too, Mistic had a positive effect and significantly increased the yields obtained for all the 12 IMPs produced in *E. coli*. In a recent report, Leviatan et al. [102] presented the use of two hydrophilic bacterial proteins, YaiN and YbeL, for membrane targeting of cargo proteins and compared these fusions tags with Mistic. The yields obtained were equivalent, or even better in one case than with Mistic, but the approach used was combinatorial, testing 8 different constructs for each target protein to find the best combination. This was not suitable for use here. In any case, given the good results obtained here and elsewhere with Mistic, we consider that the strategy applied here was more efficient and less laborious. Interestingly, when Mistic was fused to the three peripheral MPs in this study, it had a negative effect, significantly reducing the yields of protein recovered with the plasma membrane. This leads us to hypothesise that Mistic might not just address the proteins to the membrane, but actually force them into the lipid bilayer. Since peripheral proteins are quite hydrophilic and interact with the membrane mainly through electrostatic interactions, being forced into the hydrophobic environment of the membrane, because of fusion to Mistic, could thus be unfavourable.

### *L. lactis*, an efficient and valuable alternative to *E. coli*

In terms of success rate and protein yields, *L. lactis* also gave good results. Together with *E. coli*, it proved to be an adequate system for the expression of *A. thaliana* MPs (Figure 6B) but, in this case, without requiring fusion to Mistic-type tags. We believe that this is due to the fact that both *L. lactis* and *A. thaliana* have very similar GC-content in their genome, as well as similar amounts and types of glycolipids in their membranes [54]. Moreover, even produced at very low levels in *L. lactis* (around 0.2% of TMP), some recombinant MPs were active in this system [54]. The difference in protein yields obtained with the two bacteria could be explained by the limited capacity of *L. lactis* to accumulate branched-chain amino acids, thus limiting overexpression in this host [103]. *L. lactis* appears to be complementary to *E. coli*: the 17 MPs expressed (including all the MPs from *A. thaliana*), could be produced in at least one of these two bacteria. The three proteins that failed to express in these bacteria (MraY, α2δ subunit and NIS) also failed in all the other systems tested. For the less hydrophobic MPs, the two systems were equivalent: all the peripheral proteins and those containing a single predicted TM were produced in both bacteria. In contrast, MPs with higher numbers of TMs (UCP1, AAC and PHT1;4) were only produced in *E. coli*, whereas AtHMA3 and AtHMA4, as well as AtHMA1 (without the Mistic tag), were only detectable in *L. lactis*. Thus, *L. lactis* is an efficient expression system and it should be considered as an alternative when overexpression fails in *E. coli*.

### The benefits of homologous and eukaryotic expression

With heterologous protein expression the recombinant protein produced does not always truly resemble the native protein. Conditions that produce the largest amount of protein do not necessarily generate functional proteins [7], [29], [104]–[106] and, in many cases, proteins are only functional after post-translational modification, such as through glycosylation and formation of disulfide bonds. Although several prokaryotic strains have been developed to overcome some of these hurdles (e.g. *E. coli* trxB mutants or *E. coli* glycosylation enabled mutants [107]), eukaryotic systems are sometimes necessary. Three eukaryotic hosts were selected in this project (*A. thaliana, N. benthamiana,* Sf9). *A. thaliana* enabled homologous expression of several proteins (9 out of 20 originate from this organism) and it allowed us to show that the protein AAC was correctly targeted to mitochondria (Figure 4). Different approaches to protein production were used in the two plant systems. In *A. thaliana* stable cell lines were generated, while in *N. Benthamiana* transient agro-infiltration was used. Equivalent yields were obtained for proteins expressed in both systems, but more targets were produced in *A. thaliana*. However, the faster turnaround time with transient agro-infiltration is a great advantage, and facilitates screening for optimal production conditions (e.g. light intensity). In Sf9 cells, eight proteins were correctly overexpressed, and all five well-expressed proteins were of eukaryotic origin. Nonetheless the rat α2δ subunit and human CXCR4 proteins were not expressed at all, and human CCR5 was produced in an aggregated form. This suggests that insect cells are not necessarily able to handle mammalian proteins properly.

Based on these results, and those discussed above for bacterial protein expression, prokaryotic and eukaryotic systems are complementary. Even though compared to *E. coli*, the expression levels in the other systems are generally lower, a significant number of targets could however be expressed, proving that *L. lactis, R. sphaeroides, A. thaliana* or *N. benthamiana* are valuable alternatives to more conventional expression hosts and can be considered for expression of membrane proteins. Most of these systems can be rather easily implemented in a laboratory. *L. lactis* and *R. sphaeroides* systems require similar handling procedures and instrumentations that are used for *E. coli*. Many commercial alternatives are available for expression in insect cells and the protocols are well established. However, insect cells culture medium is three to four times more expensive than *E. coli* medium. The agroinfiltration procedure required for expression in *N. benthamiana* is rather simple and well described in the literature [62], [108]. Plant culturing requires an illuminated growth chamber, but doesn't cause major problems and a few days training in an expert laboratory should be sufficient to learn the necessary techniques. On the other hand, the procedures described here for expression in *A. thaliana* and the obtention of stable lines requires much more time and expertise, and one should consider a collaboration with an appropriate laboratory.

Protein activity also needs to be evaluated to choose the appropriate expression system for further functional studies.

### Towards functional characterisation of the recombinant proteins produced

Overexpression of membrane proteins is a challenge in itself. Many groups aiming to characterise one of these difficult membrane proteins, must first test the efficiency of various expression systems before any functional characterisation can be performed. In this study, we intentionally focused on the expression yields of the 20 proteins tested, and on the ability of the six different hosts to produce our target proteins. Indeed, a good expression yield is a prerequisite for most biochemical and biophysical experiments as it more or less determines the final purity, concentration in solution, amount of protein available, cost of production, etc. In addition, in some cases, producing enough protein, whether functional or not, is a goal in itself. These proteins can be used to develop precious tools such as, for example, antibodies that usually work better than antibodies raised against shorter synthetic peptides. In this study, we also chose to study quite a diverse range of proteins (size, hydrophobicity, origin) with different known or predicted activities, even though, for many of them, no functional assays had yet been performed. For most of these proteins, functional characterisation represents a stand-alone project and it would not be possible to perform it for the 120 host/protein combinations described here.

In parallel studies, the functionality of some of the proteins produced during the present study has been analysed in one or more expression hosts. Thus, ceQORH proved to be active when produced in either *E. coli* or *L. lactis*, but its specific activity was higher in *L. lactis,* and the protein could be purified without difficulty using either the His-tag or the *Strep*-tag II [54]. The activity of AAC, produced in *E. coli*, was also tested, it was found to transport radioactive ATP, and was also sensitive to variations in NaCl concentration [77]. The functionality of NTT1 in the two bacterial systems (*E. coli* and *L. lactis*) and the influence of fusion to Mistic fusion (in *E. coli*) were also analysed. This protein was active in both in *E. coli* and *L. lactis* [54], [76]. When produced in *E. coli*, the protein could be purified to homogeneity and, by optimising growth conditions, the Mistic fusion led to a 16-fold increase in protein yield. Although the transport activity of the fusion protein was impaired in *E. coli* membranes, cleavage of the Mistic moiety *in vivo* delivered a functional transporter, proving that the Mistic strategy is a valuable approach [76]. Last but not least, of the four plant heavy metal ATPases tested in *L lactis*, two were efficiently produced and purified from this host [54]. This system was also recently used to perform the first biochemical characterisation of a plant copper ATPase [109].

### Conclusion

This study compared several approaches for the overexpression of a variety of recombinant membrane proteins in six expression hosts. Our success rate was high, with 17 out of 20 proteins tested expressed in at least one system, but large and very hydrophobic proteins remained however hard to express whatever the host used. It is therefore important to keep in mind that optimisation of expression conditions can greatly improve the yields of protein produced and it should be thoroughly undertaken after selecting the host. If a first screen fails to identify an appropriate expression system for a given target protein, optimisation of growth conditions could then be attempted in one of the most successful hosts presented here, e.g. *E. coli* or *L. lactis*.

The different systems present various advantages. Very good yields (several mg protein/L culture) could be obtained with *E. coli*, especially when Mistic fusions were used. But it is important to gather topological information on the target before fusing it to Mistic, as fusion with peripheral MPs was detrimental for the three proteins tested. Therefore, Mistic should only be considered as an aide to correct membrane targeting of IMPs. *L. lactis* was an appropriate host for the expression of plant MPs, as well as a good alternative to *E. coli* when expression fails in this system. We demonstrated homologous expression in *A. thaliana* to be beneficial, as it allows the investigation of subcellular targeting (as for ACC here). The baculovirus system was less efficient than *E. coli* or *L. lactis*, both in terms of number of expressed proteins and quantity of protein produced, but is nonetheless a good system (eight proteins expressed, five with a reasonable yield, out of 18 successfully cloned candidates). Moreover, insect cells appear to be more useful for the production of functional proteins with specific post-translational modifications, as are the other eukaryotic hosts: *A. thaliana* and *N. benthamiana*.

In this work, we have developed a certain number of methods to increase the throughput and rationalise the screening of MP overexpression in both prokaryotic and eukaryotic systems. This systematic approach was efficient since less conventional expression systems proved to be valuable alternatives and, as discussed above, besides *A. thaliana* all other systems can be rather easily implemented in other laboratories. We believe that the evaluation of expression systems presented here is a useful starting guide for biologists aiming to produce their favourite membrane protein in amounts compatible with further biochemical and structural characterisation.

## Supporting Information

Figure S1 **The successive cloning steps in the Gateway technology.** The Gateway Technology uses the λ recombination system to facilitate transfer of heterologous DNA sequences (flanked by modified *att* sites) between vectors. BP Reaction: Facilitates recombination of an *att*B-PCR product with an *att*P-containing donor vector to create an *att*L-containing entry clone. This reaction is catalysed by BP Clonase. LR Reaction: Facilitates recombination of an *att*L-containing entry clone with an *att*R-containing destination vector to create an *att*B-containing expression clone. This reaction is catalysed by LR Clonase.
(TIF)

Figure S2 **Influence of the number of TMs on the expression in the different systems.** The bars represent the percentage of positively expressed proteins in each expression host for a given category. Light blue: *E. coli*; Red: *L. lactis*; Yellow: *R. Sphaeroides*; Green: *A. thaliana*; Dark blue: *N. benthamiana*, Orange: insect cells. Global expression represents the percentage of positively expressed proteins in all expression hosts for a given category.
(TIF)

Figure S3 **Influence of the protein size on the expression in the different systems.** The bars represent the percentage of positively expressed proteins in each expression host for a given category. Light blue: *E. coli*; Red: *L. lactis*; Yellow: *R. Sphaeroides*; Green: *A. thaliana*; Dark blue: *N. benthamiana*, Orange: insect cells. Global expression represents the percentage of positively expressed proteins in all expression hosts for a given category.
(TIF)
