# Supplementary figures and images for: Heterologous Expression of Membrane Proteins: Choosing the Appropriate Host
Source: PLoS One. 2011 Dec 21;6(12):e29191. doi: 10.1371/journal.pone.0029191 (PMC3244453; doi:10.1371/journal.pone.0029191)

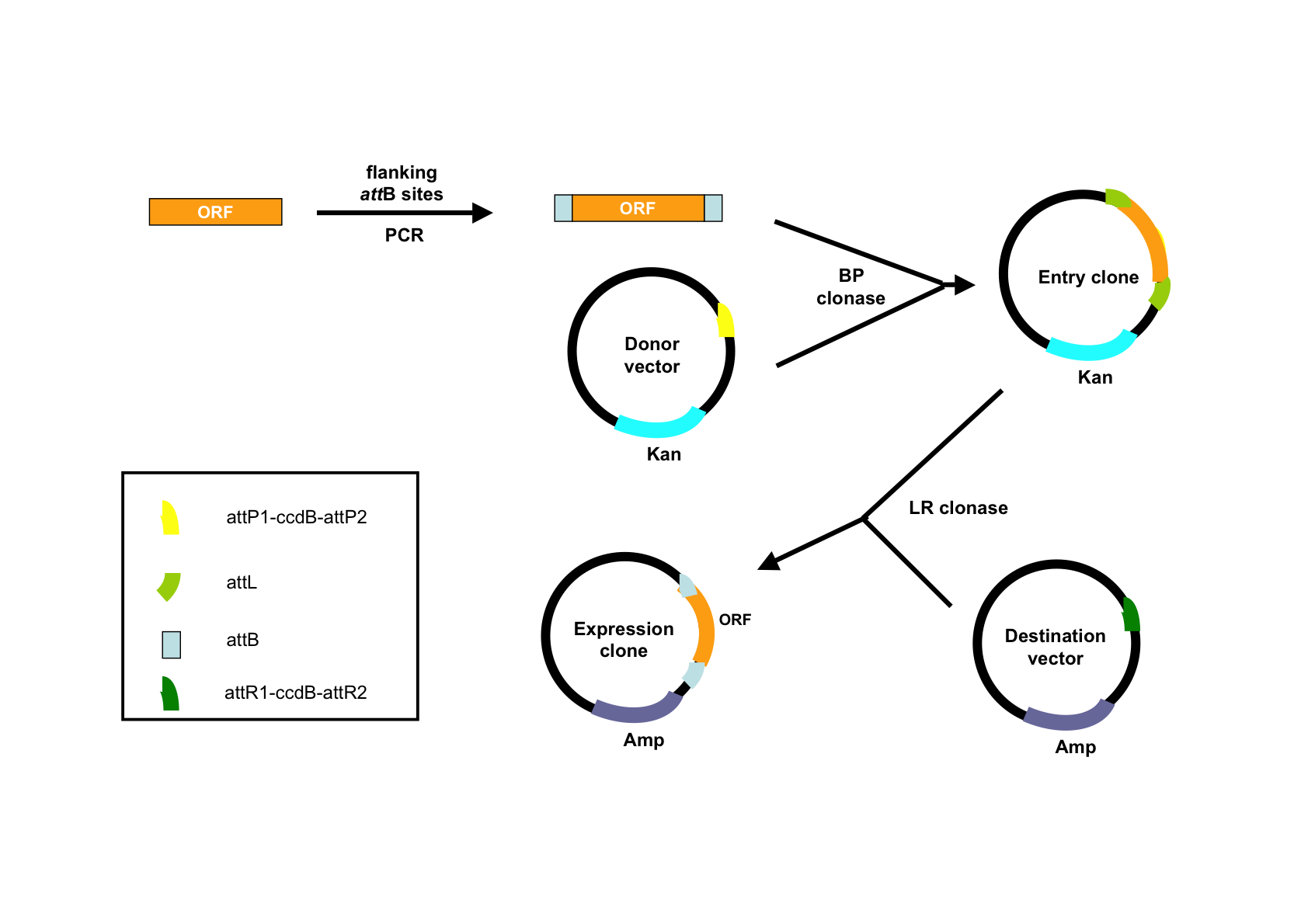

Supplement: Figure S1 — The successive cloning steps in the Gateway technology. The Gateway Technology uses the λ recombination system to facilitate transfer of heterologous DNA sequences (flanked by modified att sites) between vectors. BP Reaction: Facilitates recombination of an attB-PCR product with an attP-containing donor vector to create an attL-containing entry clone. This reaction is catalysed by BP Clonase. LR Reaction: Facilitates recombination of an attL-containing entry clone with an attR-containing destination vector to create an attB-containing expression clone. This reaction is catalysed by LR Clonase. (TIF) [file pone.0029191.s001.tif]

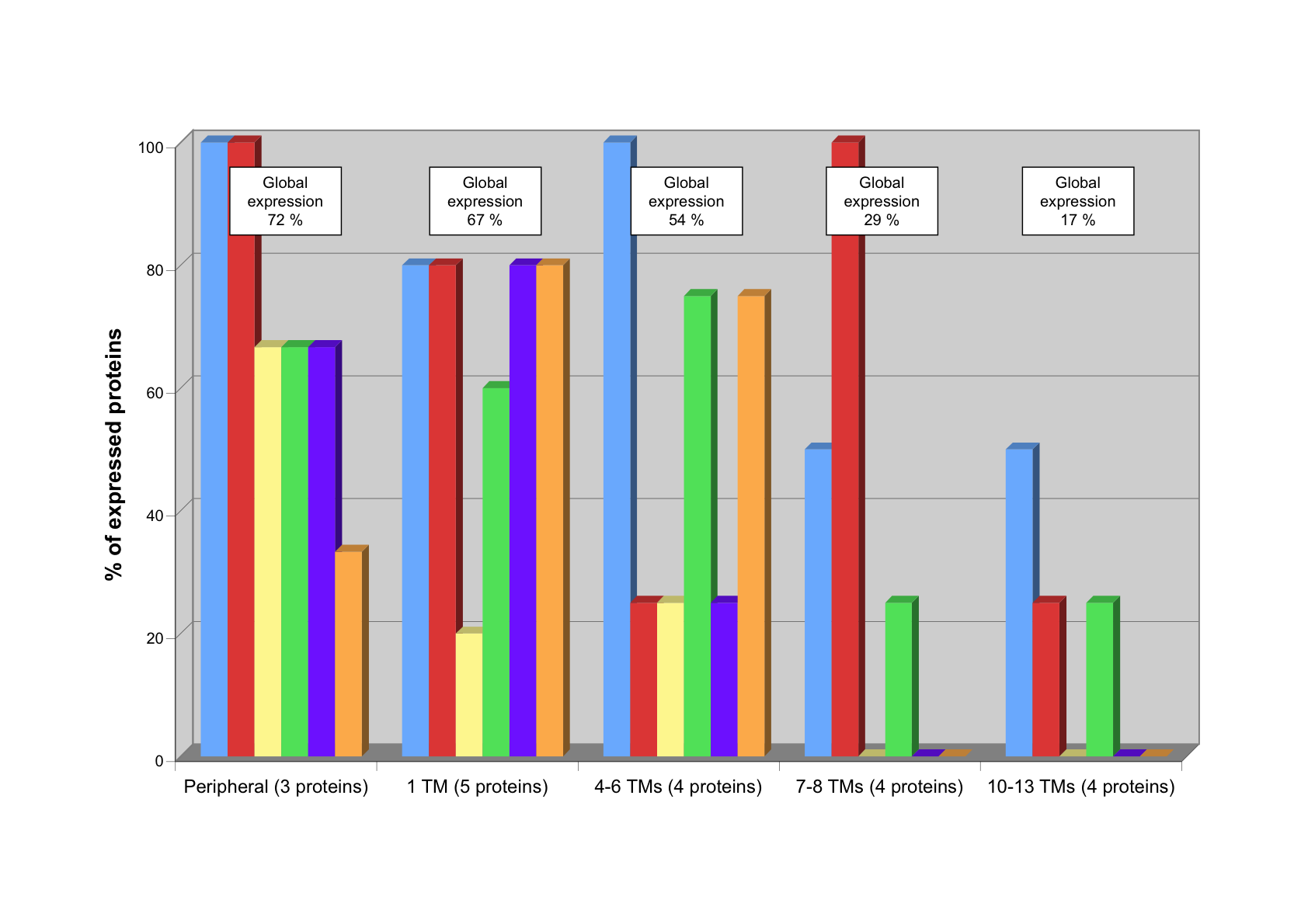

Supplement: Figure S2 — Influence of the number of TMs on the expression in the different systems. The bars represent the percentage of positively expressed proteins in each expression host for a given category. Light blue: E. coli; Red: L. lactis; Yellow: R. Sphaeroides; Green: A. thaliana; Dark blue: N. benthamiana, Orange: insect cells. Global expression represents the percentage of positively expressed proteins in all expression hosts for a given category. (TIF) [file pone.0029191.s002.tif]

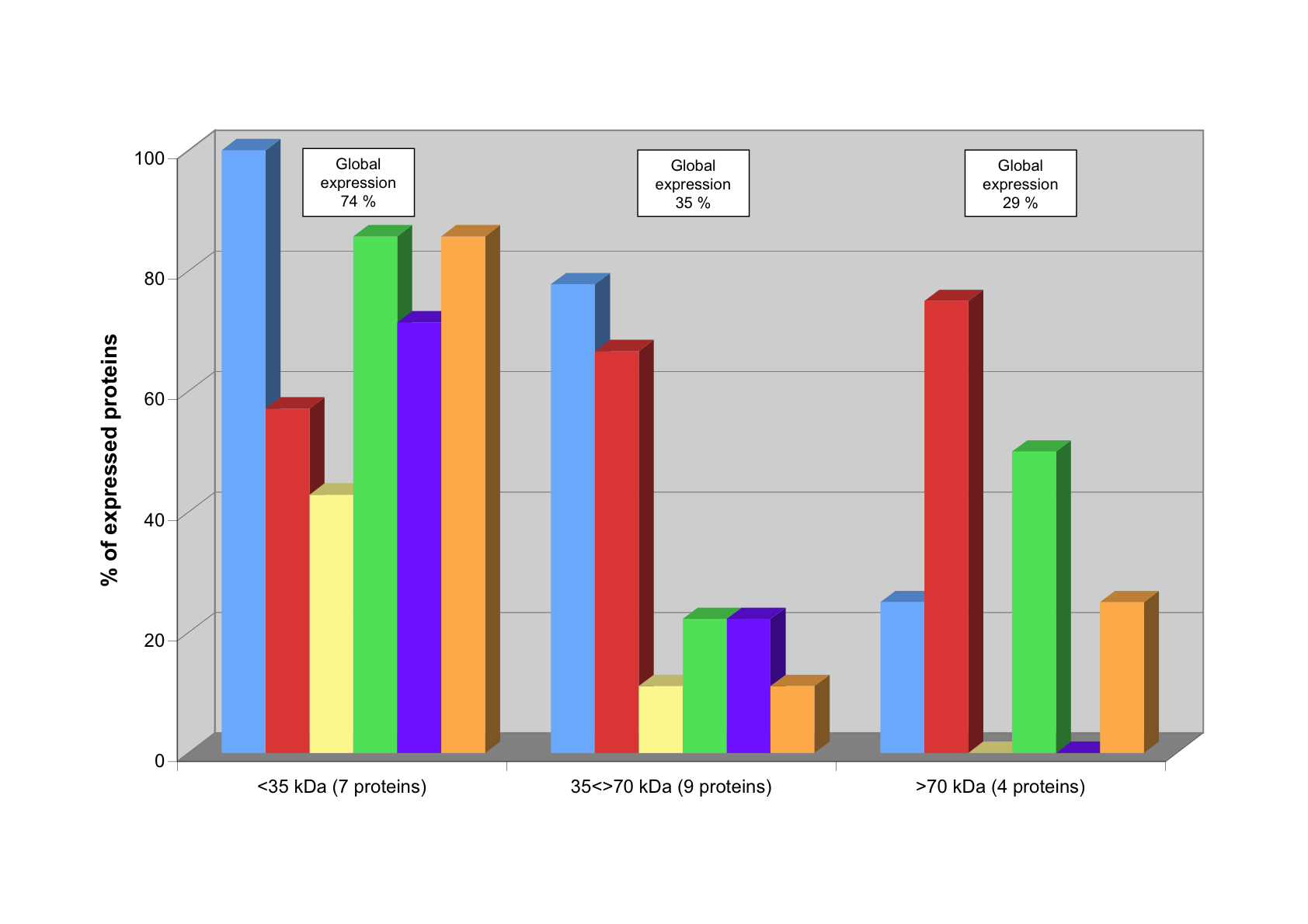

Supplement: Figure S3 — Influence of the protein size on the expression in the different systems. The bars represent the percentage of positively expressed proteins in each expression host for a given category. Light blue: E. coli; Red: L. lactis; Yellow: R. Sphaeroides; Green: A. thaliana; Dark blue: N. benthamiana, Orange: insect cells. Global expression represents the percentage of positively expressed proteins in all expression hosts for a given category. (TIF) [file pone.0029191.s003.tif]
